# A Self-Regulation-Based eHealth Intervention to Promote a Healthy Lifestyle: Investigating User and Website Characteristics Related to Attrition

**DOI:** 10.2196/jmir.7277

**Published:** 2017-07-11

**Authors:** Celien Van der Mispel, Louise Poppe, Geert Crombez, Maïté Verloigne, Ilse De Bourdeaudhuij

**Affiliations:** ^1^ Research Group Physical Activity and Health Department of Movement and Sports Sciences Ghent University Ghent Belgium; ^2^ Ghent Health Psychology Lab Department of Experimental-Clinical and Health Psychology Ghent University Ghent Belgium

**Keywords:** physical activity, healthy diet, eHealth, attrition, self-regulation

## Abstract

**Background:**

eHealth interventions can reach large populations and are effective in increasing physical activity (PA) and fruit and vegetable intake. Nevertheless, the effects of eHealth interventions are overshadowed by high attrition rates. Examining more closely when users decide to leave the intervention can help eHealth developers to make informed decisions about which intervention components should be reshaped or simply removed. Investigating which users are more likely to quit an intervention can inform developers about whether and how their intervention should be adapted to specific subgroups of users.

**Objective:**

This study investigated the pattern of attrition in a Web-based intervention to increase PA, fruit, and vegetable intake. The first aim was to describe attrition rates according to different self-regulation components. A second aim was to investigate whether certain user characteristics are predictors for start session completion, returning to a follow-up session and intervention completion.

**Methods:**

The sample consisted of 549 adults who participated in an online intervention, based on self-regulation theory, to promote PA and fruit and vegetable intake, called “MyPlan 1.0.” Using descriptive analysis, attrition was explored per self-regulation component (eg, action planning and coping planning). To identify which user characteristics predict completion, logistic regression analyses were conducted.

**Results:**

At the end of the intervention program, there was an attrition rate of 78.2% (330/422). Attrition rates were very similar for the different self-regulation components. However, attrition levels were higher for the fulfillment of questionnaires (eg, to generate tailored feedback) than for the more interactive components. The highest amount of attrition could be observed when people were asked to make their own action plan. There were no significant predictors for first session completion. Yet, two subgroups had a lower chance to complete the intervention, namely male users (OR: 2.24, 95% CI=1.23-4.08) and younger adults (OR: 1.02, 95% CI=1.00-1.04). Furthermore, younger adults were less likely to return to the website for the first follow-up after one week (OR: 1.03, 95% CI=1.01-1.04).

**Conclusions:**

This study informs us that eHealth interventions should avoid the use of extensive questionnaires and that users should be provided with a rationale for several components (eg, making an action plan and completing questions). Furthermore, future interventions should focus first on motivating users for the behavior change before guiding them through action planning. Though, this study provides no evidence for removal of one of the self-regulation techniques based on attrition rates. Finally, strong efforts are needed to motivate male users and younger adults to complete eHealth interventions.

## Introduction

eHealth is defined as “the use of information and communications technology, especially the Internet, to improve or enable health and health care” [1]. Compared with traditional face-to-face health interventions, eHealth interventions have the potential to reach large populations in a time-efficient way. Furthermore, these interventions can be tailored to users’ needs and have shown to be effective in changing health behavior, such as increasing physical activity (PA) [2-4] and fruit and vegetable intake [5]. Despite the promising results, the effects of eHealth interventions are often plagued by high attrition rates. With attrition rates reaching 60-80%, loss of participants during the intervention seems to be the rule rather than the exception in eHealth research [6]. Possible effects of the intervention may then be compromised due to low exposure to the intervention content [7]. That way, high attrition rates are a threat for the internal and external validity of the intervention results [8].

According to Eysenbach [9], 2 types of attrition in eHealth can be identified. The first type, called nonusage attrition, refers to attrition from the intervention and occurs when participants stop using the eHealth intervention. This problem can arise at any given moment, for example, when participants do not complete a website session or when they do not return to the website anymore. The second type of attrition refers to participants withdrawing from the study itself. The phenomenon of participants not returning for follow-up assessment sessions is described by the term dropout attrition. Both types of attrition can challenge eHealth research. Nonusage attrition can undermine the potential effect of an intervention (due to low exposure to the intervention content), whereas dropout attrition might influence the power and the results of the study that evaluates the intervention [10].

Investigating patterns of nonusage attrition can provide valuable information for the development of eHealth interventions [9]. By examining when users discontinue the intervention, possible obstacles can be identified. Researchers often describe attrition rates at the end of the intervention and investigate predictors of intervention completion [8,11-13]. However, attrition can occur at all stages of the intervention. To our knowledge, no study has examined nonusage attrition early on in the intervention, that is, during an intervention program. Examining more closely when users decide to leave the intervention can help eHealth developers to make informed decisions about which parts or components of the intervention tool should be redesigned or simply removed. Attrition should thus be investigated as a function of different meaningful intervention components.

Many eHealth interventions require participants to fill out questionnaires for either providing tailored feedback or research purposes. However, it is it not known whether this affects the attrition rates of the eHealth program. Furthermore, self-regulation techniques (eg, action planning, coping planning, and monitoring) play an important role in many behavior change theories [14-16] and are therefore often implemented in eHealth interventions (eg, see [17-19]). These techniques are theory-based and elicit behavior change [20]. However, there is a lack of research that investigates whether participants easily adopt using these techniques, or rather whether the implementation of these techniques in eHealth interventions is related to attrition. Thus, identifying critical components in an intervention, that is, moments during which nonusage attrition peaked, can provide useful information.

Of further importance is to identify who is less likely to complete the eHealth intervention. For example, research shows that the utilization of eHealth tools depends upon the age of its users, with younger adults being more likely to show higher levels of nonusage attrition than older adults [6,21,22]. Also, men and users with a lower level of education have higher chances to show low levels of eHealth utilization [21,23,24]. However, to our knowledge, attrition according to age, sex, or education level has not been thoroughly investigated in self-regulation-based eHealth interventions. Finally, body mass index (BMI) could be predictive for the completion of eHealth interventions, although previous research on the predictive value of BMI in completing weight-loss interventions shows inconsistent results [25-29]. Identifying groups of users who are more likely to quit a Web-based program can inform developers about whether and how an intervention should be adapted to specific subgroups of users. Further research can then help us define the unaddressed needs of these subgroups. By doing so, the reach and effectiveness of future eHealth interventions can be ameliorated.

This paper investigates nonusage attrition from the eHealth intervention “MyPlan 1.0”. MyPlan 1.0 is a website that aims to increase PA and the intake of fruit and vegetables in the adult population [30]. This intervention is based on self-regulation theory [14], which is the process of goal selection, goal pursuit, and goal maintenance. MyPlan 1.0 thus includes different self-regulation techniques that can be investigated for their likelihood of increasing or decreasing attrition. The first technique included in MyPlan 1.0 is providing tailored feedback. Therefore, participants complete questionnaires regarding their current behavior and receive advice that compares their behavior with the guidelines and provides examples on how they could improve their behavior. A second technique is coping planning, in which users identify possible obstacles and solutions. The program also contains action planning. Here users define what they want to achieve and when and where exactly they are planning to do so. Also included is self-monitoring of behavior, which is facilitated by prompting users to reflect upon how they will keep track of their behavior (eg, in their diary or via cellphone). Finally, the use of social support is encouraged by providing users the opportunity to email their personal plan to a friend or family member. More information on how the techniques were implemented in the website is described in the Multimedia Appendix 1. These techniques were carefully selected based on their potential effectiveness, described in the current literature. Previous research demonstrated the effectiveness of MyPlan 1.0 as a whole to increase PA and the consumption of fruit and vegetables in adults [31-33]. However, like many eHealth interventions, MyPlan 1.0 is challenged by high rates of attrition: at the end of the intervention a loss of 64.0% (235/367) of the participants was observed [32]. In this program, participants that caused nonusage attrition were automatically causing dropout attrition since participants completed all measures in the Web-based program. In this article, we focus on nonusage attrition and aim to identify the components that make people stop using an intervention in which they initially showed interest.

The aim of this paper is two-fold. First, we aim to identify critical moments of attrition in the eHealth intervention MyPlan 1.0 using an explorative and quantitative approach. Therefore, we will describe the rates of website utilization according to the different self-regulation-based intervention components (namely providing feedback, action planning, coping planning, self-monitoring, and social support) and the general components (namely filling in demographic information and filling in a questionnaire). This may help us understand which components in an eHealth intervention discourage users to continue with the program. For this aim, we will also report the usage half-life of MyPlan 1.0, which is the moment where 50% of the users have stopped using the tool [9]. Our second aim is to investigate if certain user characteristics (ie, sex, education, age, and BMI) are predictors of start session completion, returning to a follow-up session, and intervention completion. This may provide information about whether the intervention distinguishes between certain subpopulations of users.

## Methods

### Participants and Design

The sample consisted of adults who participated in a Web-based intervention to promote a healthy lifestyle, called MyPlan 1.0, from November 2014 to September 2016. Participants were recruited via the general practice setting. Both researchers in the waiting room and general practitioners provided the participants with a flyer that directed them to the intervention website. There were also tablets available in the waiting room, where participants could start to fill in the intervention program. When they were not able to finish the program in the waiting room, they received a link to complete the intervention program at home. The inclusion criterion was a minimum age of 18 years. All data entered by participants were, just as the information about website use, collected and stored in LimeSurvey (LimeSurvey Project Hamburg, Germany). Participants did not receive any kind of incentive. The study was approved by the Ghent University Hospital Ethics Committee.

### Intervention

**Figure 1:**
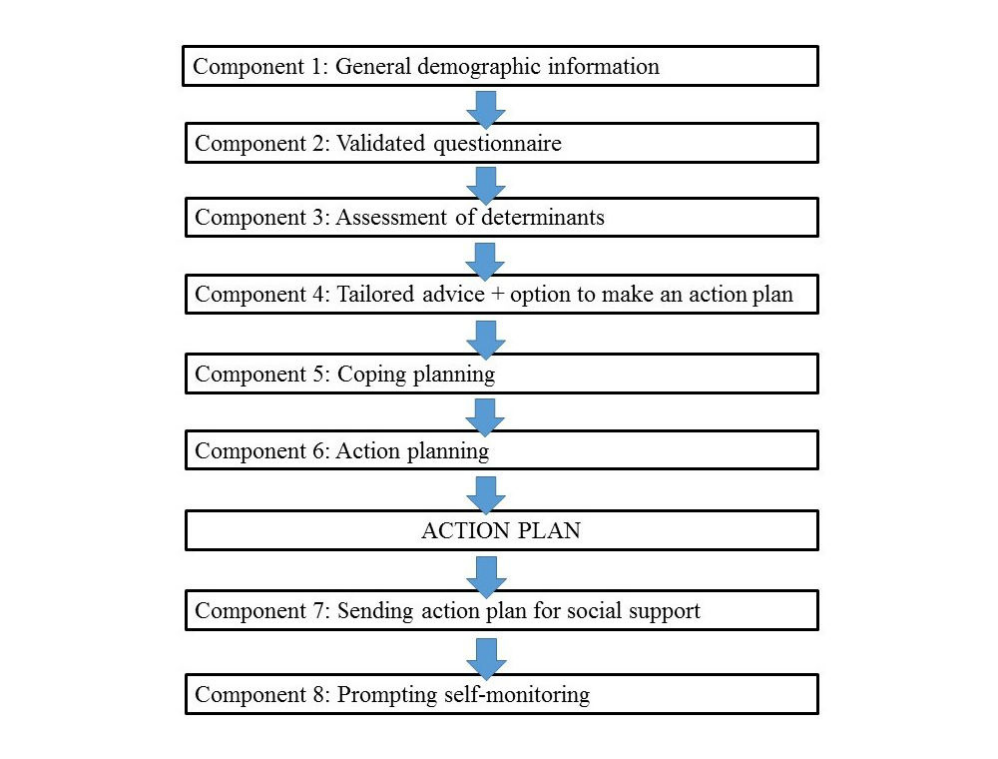
Overview of the start session components.

The Web-based intervention website MyPlan 1.0 was developed using the intervention mapping protocol [30] and has proven to be effective and feasible [32]. The intervention targets behavior change in three domains: PA, fruit intake, and vegetable intake. In a first step, participants choose which behavior they prefer to change. Thereafter, the structure of the intervention is identical for the three behaviors. The intervention consists of 3 sessions: one start session, and two follow-up sessions. In the start session, participants are making personal health action plans for the first time. After 1 week, they get an invitation by email to complete the second session of the intervention (follow-up 1, FU1). In this follow-up session they get feedback on their behavior change and can choose to keep or adapt their personal action plan according to their success or failure. One month after the first session, the third and last session (follow-up 2, FU2) is activated, in which they evaluate their behavior change a second time. The intervention is based upon self-regulation theory [14,34] and guides participants in their behavior change through different mandatory components based on self-regulation techniques (namely providing feedback, action planning, coping planning, self-monitoring, and social support). Figure 1 illustrates the flow of the start session, in which all self-regulation techniques are incorporated. Within this first session, participants start by filling in general demographic information. Thereafter, they complete a validated questionnaire regarding the chosen behavior (International Physical Activity Questionnaire [IPAQ] [35]; The Flemish Fruit Test and Vegetable Test, [36]) and get tailored feedback on their current level of PA or fruit or vegetable intake. For study purposes, participants also fill out an assessment of determinants of behavior change such as self-efficacy and motivation. After the tailored advice, participants can choose to make an action plan or to leave the website. In order to make an action plan, participants complete a coping planning and an action planning component, respectively. In the coping planning component, they identify possible difficulties and make a plan to overcome these barriers. In the action planning component, they are guided to define where, when, and in which way they would like to be physically active or eat more fruit or vegetables. Participants also get the option to state implementation intentions [37,38], that is, to formulate an if-then plan (eg, if I come home from work, I go walking in the neighborhood for half an hour). This information is collected and shown in a comprehensive action plan. Participants can choose to send their action plan to family or friends in order to get social support. At the end, the website asks participants how they are going to keep track of their activity or fruit or vegetable intake in order to prompt self-monitoring of behavior change.

### Measures and Statistical Analysis

#### Description of the Nonusage Attrition Pattern

To analyze the nonusage attrition during the intervention (aim 1), the start session was divided into 8 components according to the different self-regulation techniques and the general information part, as described previously and depicted in Figure 1. If the last question of the component was answered or the last choice option was filled in, the component was considered as completed. If not, nonusage attrition occurred during that specific component. Attrition as a function of the different components was described in terms of absolute and relative numbers. Critical components during the follow-up sessions were not analyzed because the self-regulation techniques included in these sessions were very similar to the ones in the start session.

#### Predictors of Intervention Completion

Besides nonusage attrition in the start session, predictors (ie, users’ demographic information) of intervention completion were also investigated (aim 2). Demographic characteristics were obtained from the answers given in the start session of the website intervention. Demographic measures included sex, educational level, age, height, and weight. Regarding educational level, a college degree was considered as high educational level, whereas no education, primary school, and secondary school were considered as low educational level. BMI was calculated by dividing weight (in kilogram) by height (in meter) squared. Participants were classified as not overweight if they had a BMI under 25 kg/m² and as overweight if their BMI was 25 kg/m² or higher.

Completion was defined as follows: if the last question of the start session was filled in, the session was considered as completed. Returning to the website was defined as accessing FU1, or more specifically, “filling in the first question of FU1.” If the last question of the last session (FU2) was filled in, the whole intervention was considered as completed. To investigate aim 2, three logistic regression analyses were conducted in SPSS version 23 (IBM Corporation): (1) to identify predictors of start session completion, (2) to investigate predictors of a first return to the website after start session completion (ie, accessing FU1), and (3) to examine predictors of FU2 completion (ie, intervention completion). After checking for multicollinearity, all demographic variables (ie, sex, educational level, age, and BMI) were entered together into the regression as possible predictors. The level of significance was set at *P*<.05.

## Results

### Participant Characteristics

In total, 549 adults visited the intervention website and were therefore defined as “potential users”. However, 127 of them only visited the home page and did not register (ie, fill in their name and email address). They were excluded from the analyses since no information about them was available. The remaining 422 were considered as “actual users”; 39.1% (165/422) of them chose to focus on PA, 41.0% (173/422) on fruit intake, and 19.9% (84/422) on vegetable intake. All participants that registered were included in the study, although it has to be noted that some people registered but did not complete (all) demographic measures.

An overview of the participant characteristics can be found in Table 1. In the total sample, 55.7% (235/422) were female and 28.2% (119/422) were male. Furthermore, 41.7% (176/422) of the people had a low educational level, whereas 42.2% (178/422) had a high educational level. The mean age of the sample was 43.92 years (SD 14.23), ranging from 18-81 years. Finally, 48.3% (204/422) of the sample was overweight, whereas 48.6% (205/422) had a normal weight. The mean BMI was 25.96 (SD 5.39) kg/m².

**Table 1:** Overview of participant’s demographic characteristics (N=422).

Characteristics		n (%)	Mean (SD)
**Sex**			
	Male	119 (28.2)	
	Female	235 (55.7)	
	Missing	68 (16.1)	
**Education**			
	Low	176 (41.7)	
	High	178 (42.2)	
	Missing	68 (16.1)	
**BMI (kg/m²)**			25.96 (5.39)
	Overweight	204 (48.3)	
	Not overweight	205 (48.6)	
	Missing	13 (3.1)	
**Age (years)**			43.92 (14.23)
	Missing	68	

### Description of the Nonusage Attrition Pattern

In total, 55.7% (235/422) of the participants completed the start session. Only 43.1% (182/422) of the total sample returned to the first follow-up session. Therefore, the usage half-life is situated between the start session and FU1. Of the total sample, 21.8% (92/422) completed FU2. Hence, at the end of the intervention program, there was a nonusage attrition rate of 78.2% (330/422).

**Figure 2:**
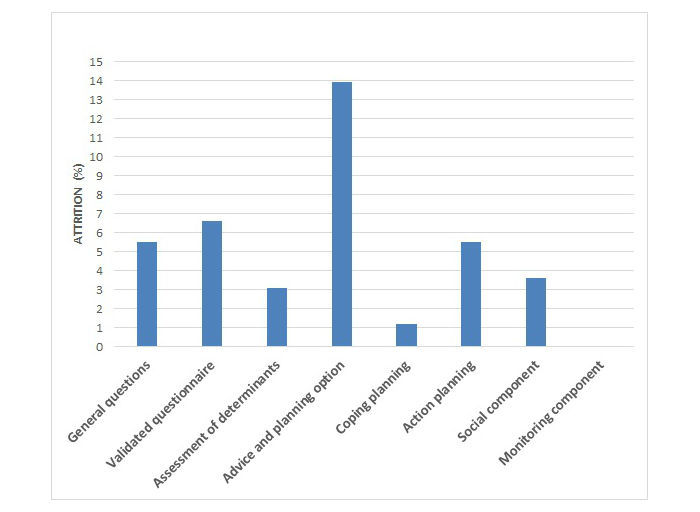
Attrition percentage per website component in the start session of the physical activity module.

**Figure 3:**
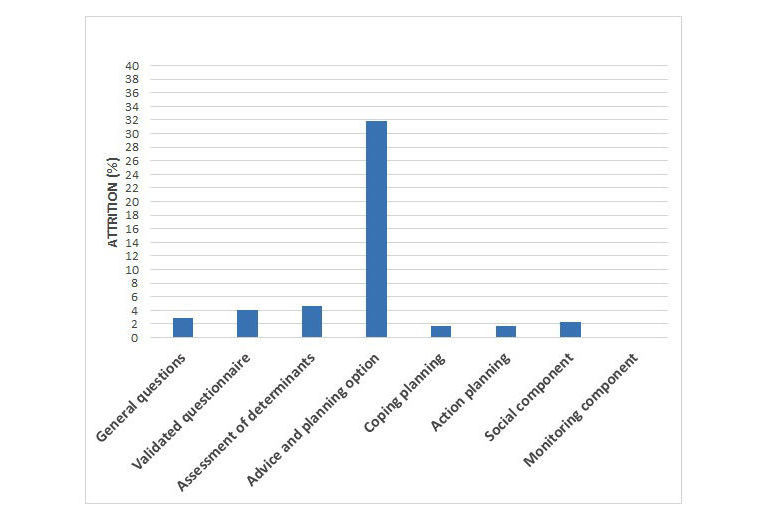
Attrition percentage per website component in the start session of the fruit module.

**Figure 4:**
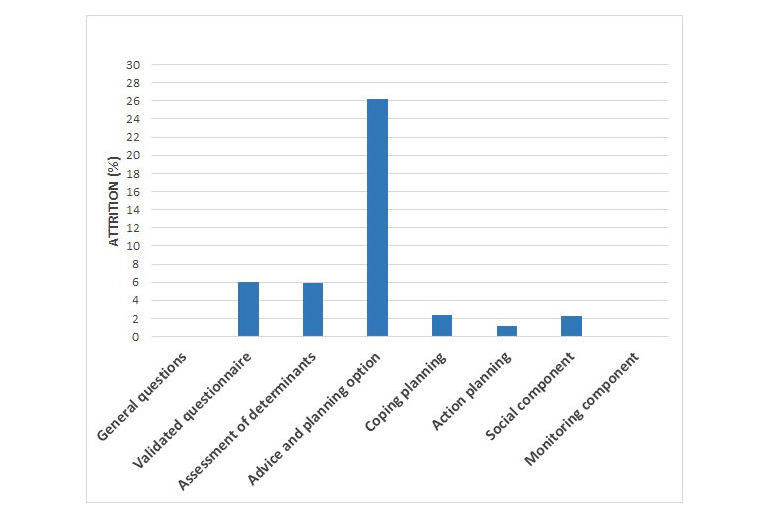
Attrition percentage per website component in the start session of the vegetable module.

To identify components (eg, action planning and coping planning) in which nonusage attrition is the highest, the start session was divided into eight components, as described in the methods section. The critical moments were defined separately for the three target behaviors (PA, fruit intake, and vegetable intake) in order to get a more detailed insight in possible obstacles during intervention fulfilment. The extent to which attrition occurred per component can be found in Table 2. Results are also visualized in Figures 2-4 for the PA, fruit, and vegetable module, respectively. All components show attrition rates of less than 5%. The only component for which attrition rates are higher than 5% in all three modules is the advice and planning option.

**Table 2:** Attrition rates per website component.

Session	Website component	Physical activity (n=165)		Fruit intake (n=173)		Vegetable intake (n=84)	
		n	% (cumulative %)	n	% (cumulative %)	n	% (cumulative %)
**Start session**		65	39.4 (39.4)	85	49.1 (49.1)	37	44.0 (44.0)
	General questions	9	5.5 (5.5)	5	2.9 (2.9)	0	0 (0)
	Validated questionnaire	11	6.6 (12.1)	7	4.0 (6.9)	5	6.0 (6.0)
	Assessment of determinants	5	3.1 (15.2)	8	4.7 (11.6)	5	5.9 (11.9)
	Advice and planning option	23	13.9 (29.1)	55	31.8 (43.4)	22	26.2 (38.1)
	Coping planning	2	1.2 (30.3)	3	1.7 (45.5)	2	2.4 (40.5)
	Action planning	9	5.5 (35.8)	3	1.7 (46.8)	1	1.2 (41.7)
	Social component	6	3.6 (39.4)	4	2.3 (49.1)	2	2.3 (44)
	Monitoring component	0	0 (39.4)	0	0 (49.1)	0	0 (44)
							
**Follow-up 1**		57	34.5 (73.9)	40	23.2 (72.3)	27	32.2 (76.2)
**Follow-up 2**		7	4.3 (78.2)	4	2.3 (74.6)	8	9.5 (85.7)

**Table 3:** Predictors for start session completion, returning after start session completion, and intervention completion.

Session		Exp (B)^a^	SE^b^	95% CI
**Start session completion**					
	Sex	1.35	0.23	0.85-2.12
	Education	1.10	0.22	0.72-1.68
	Age	1.01	0.01	0.99-1.02
	Overweight or not	1.15	0.23	0.74-1.79
**Returning to FU1^c^**					
	Sex	1.22	0.24	0.77-1.94
	Education	1.07	0.22	0.69-1.64
	Age	1.03	0.01	1.01-1.04
	Overweight or not	1.41	0.23	0.90-2.20
**Intervention completion**					
	Sex	2.24	0.31	1.23-4.08
	Education	1.15	0.26	0.69-1.92
	Age	1.02	0.01	1.00-1.04
	Overweight or not	1.28	0.27	0.75-2.16

^a^Exp(B): exponential function of the coefficient B. This indicates the odds ratio for the predictor.

^b^SE: standard error.

^c^FU1: follow-up 1.

### Predictors of Intervention Completion

There were no significant predictors for start session completion (see Table 3). However, there was one significant predictor for returning after start session completion (see Table 3). Age group significantly predicted whether participants would return to the website after 1 week (Odds ratio [OR]=1.03, 95% CI 1.01-1.04), with older participants being more likely to return than younger participants. There were two significant predictors for FU2 completion as well (see Table 3). Both age (OR=1.02, 95% CI 1.00-1.04) and sex (OR=2.24, 95% CI 1.23-4.08) could predict intervention completion, with older participants and women being more likely to complete the intervention.

## Discussion

### Principal Findings

This paper investigated both website and user characteristics related to nonusage attrition levels from a self-regulation-based eHealth tool (MyPlan 1.0). First, possible obstacles were identified by exploring attrition rates for the self-regulation techniques and general components of the start session. Second, we investigated which user characteristics predicted whether users finished the start session, returned to the website (ie, logged in for the second session), and completed the whole intervention (ie, the third session). Results show an overall attrition rate of 78.2%. Although attrition rates were similar for the various components, attrition levels were higher for filling out questionnaires (eg, to generate tailored feedback) than for the more interactive components (such as action planning, coping planning, etc). The highest amount of attrition could be observed when people were shown the advice and asked to make their own action plan. There were no significant predictors for first session completion. Yet, younger adults were less likely to return to the website for the follow-up after 1 week. Furthermore, male users and younger adults had a lower chance to complete the intervention.

A notable finding is that a large amount of users did not register when visiting the website. Previous research has already indicated that a registration procedure can be a barrier for starting an intervention [39]. This could be due to the loss of anonymity: people might be concerned about their privacy or afraid of spam mail. Providing information about the necessity to register and how personal data will be used, could overcome this problem [39]. This result further shows that not only piloting the active components (ie, behavior change techniques such as action planning), but also the more technical components (eg, registration procedure) of eHealth programs in the population of interest is very important to investigate the acceptability and feasibility of the whole intervention.

The attrition rates were similar for the various health behaviors, which may indicate that our findings are not limited to one particular behavior. Furthermore, we found that attrition levels were higher during the first components than during the later ones. This might be due to the fact that the first three components included questionnaires, whereas the latter components contained self-regulation techniques that allowed more interaction between the website and the user (eg, the user indicates possible barriers and the website offers possible solutions). Moreover, a lot of questions were added for research purposes without immediate value for the users of the intervention. Completing long questionnaires without knowing the specific purpose might have discouraged users and consequently made them stop using the intervention. Previous research already indicated that including lengthy questionnaires in an eHealth tool should be discouraged [39]. Although questionnaires are needed to enable tailored feedback, which has shown to be more effective than generic [40], the length of these questionnaires should be kept to a minimum. Furthermore, it might also be important to inform users about the necessity of providing information in order to make the tailoring possible. Tailoring could be made explicit by explaining how users’ answers shape the advice they get. Another possible explanation for higher attrition rates during the first components could be that users tend to discontinue an intervention mostly at the beginning of an intervention. When already further advanced in the intervention, users might be more motivated and have invested more, so they are less likely to quit. For example, we could observe that users who completed the first follow-up session were highly likely to complete the second follow-up session (attrition rates for FU2<10%).

The most critical moment (ie, the component for which attrition levels were the highest) occurred when users were shown the tailored advice and were asked whether they would like to create an action plan. Since previous research indicated that most users experienced the advice as personally relevant, interesting, and clear [32], we assume that users were rather discouraged by the question to make a plan than by seeing the advice. A possible explanation for attrition at this moment could be that users have gained what they needed from the intervention (eg, see [41]). From this perspective, attrition is not necessarily detrimental. When people are reaching the health norms, no intervention to change their behavior is needed. The fact that people are shown feedback on their behavior and potentially realize that they are reaching the norms might result in attrition at that moment. An additional analysis showed indeed that many of the users that were already physically active or eating enough fruit and vegetables at baseline, quitted the intervention at this point. For PA, 20 of the 113 users who met the guidelines, quitted at this moment, whereas in the fruit and vegetable module this was 28 of the 29 and 7 of the 7 users, respectively. Providing users with feedback regarding whether or not they reach the health norms can thus be beneficial as the original sample is narrowed down to a sample mainly consisting of users that the intervention aims to target.

However, other participants who did not meet the guidelines stopped using the intervention: for PA, 3 out of 32 users; for fruit, 27 out of 136 users; and for vegetables, 16 users out of 77. There are several possible reasons for attrition at this moment (ie, the choice option to make a personal action plan) in the target population. First, since the website was openly accessible, many users might not have been motivated enough to actually improve the chosen health behavior. Previous research has already indicated that people who are not motivated to change their health behavior will be reluctant to make specific plans to do so [42]. Open-access eHealth tools might attract a subgroup of users who are still ambivalent toward change (contemplators) (Stages of Change; [43]). These users are likely to explore the website without actually making specific plans for behavior change. According to the Stages of Change theory, these users should not be pushed toward immediate behavior change but provided with information and persuasive arguments to increase their motivation to change [43]. This could be implemented in eHealth interventions by giving users tailored information in relation to the stage they are in (eg, providing knowledge vs helping to plan change) and by providing the opportunity to easily return to the website, when they feel ready. Second, users might perceive the creation of an action plan as a more demanding task than answering multiple choice questions. Third, users might not have been aware of the advantages of making a specific plan to increase their PA, fruit or vegetable intake, and might have had the idea that the information and tailored advice were sufficient to put their newly elicited intentions into action. To overcome the latter two problems, it will be important that eHealth tools clearly explain why creating a specific action plan is beneficial during behavior change. Furthermore, not only highlighting the importance of creating an action plan but also communicating this component to the users in an engaging way is required. Components that cause high attrition rates should not immediately be thrown overboard, but they demand a process of reshaping. Researchers should search for a way to present theoretical components in an attractive way, for example, by minimizing the cognitive effort involved in component-specific tasks. Further qualitative research with possible users can help us understand why this component elicited high levels of attrition and can provide valuable information for reshaping the intervention.

We also explored which user characteristics predict returning to the website and completing the first and last module. We found that 2 subgroups had a lower chance to complete the intervention, namely male users and younger adults. Younger adults were also less likely to return to the website after 1 week. Male users were less likely to start with the intervention as well (28.2% male users in comparison with 55.7% female users in the sample). The phenomenon of younger users and male users being more likely to discontinue an intervention has been described as a recurring problem in eHealth [6,11,21,22]. Furthermore, previous research with MyPlan 1.0 showed that older users found the personal advice more interesting, informative, and motivating than the younger users did [32]. New opportunities to motivate younger adults and male users to use eHealth for an extended period of time need to be explored. Specifically involving these subgroups during the development of an eHealth intervention could help to make the intervention more acceptable. For example, Vandelanotte et al [44] conducted focus groups with middle-aged men regarding website and mobile-phone delivered PA and nutrition interventions and found that men are willing to use Web-based interventions provided that these interventions are quick and easy to use. Remarkably, education or BMI did not predict attrition in this eHealth intervention. This indicates that the intervention does not distinguish between low and high educated users and can be applied in an overweight population. Previous qualitative research already showed that the intervention was well accepted for high and low educated users [32].

### Strengths and Limitations

This study has several strengths. To our knowledge, this is the first study to investigate nonusage attrition during users’ first use of an eHealth intervention. Many articles have investigated attrition in eHealth but most of them focused on attrition related to the follow-up sessions [6,24]. Furthermore, this study was also the first to investigate attrition according to different website self-regulation components. Second, this study was conducted with a relatively large group of users with a balanced distribution in age, educational level, and BMI. Third, MyPlan 1.0 always offers users the possibility to log out and save their answers. So when users discontinue using the intervention because they are disturbed, they always had the possibility to continue on a later moment in time. Therefore, nonusage attrition here is most likely caused by the program itself rather than by external events.

This study has also some limitations. First, there was a disproportion in men versus women (28.2% vs 55.7%, respectively, cf. Table 1). Second, nonusage attrition was calculated based on the last mandatory question of each component. Therefore, no conclusions regarding specific questions within a certain component can be made. Third, the intervention only targeted PA, fruit intake, and vegetable intake for behavior change. More research is needed to investigate attrition in interventions targeting other behaviors (eg, smoking and weight loss). Fourth, we do not know why people stopped using the intervention during the specific intervention components. Therefore, further qualitative research might reveal why people struggle with certain components and provide insight in how the implementation of self-regulation techniques can be improved.

### Conclusions

In conclusion, this study indicates that eHealth developers should be aware that attrition already occurs during the first contact with the program and that lessons can be learned by analyzing attrition patterns. Besides investigating website characteristics, also exploring the predictive value of user characteristics is important to gain insight in the users’ needs. Combining these findings with qualitative research can help developers make informed decisions when adapting and optimizing intervention programs.

## Appendix

Multimedia Appendix 1 Implementation of the self-regulation techniques in MyPlan 1.0.

## Abbreviations

PA: physical activity
BMI: body mass index
FU1: follow-up 1
FU2: follow-up 2
IPAQ: International Physical Activity Questionnaire
