# Supplementary material for: A Self-Regulation-Based eHealth Intervention to Promote a Healthy Lifestyle: Investigating User and Website Characteristics Related to Attrition
Source: J Med Internet Res. 2017 Jul 11;19(7):e241. doi: 10.2196/jmir.7277 (PMC5527252; doi:10.2196/jmir.7277)
Supplement: Multimedia Appendix 1 [file jmir_v19i7e241_app1.pdf]

Implementation of the self-regulation techniques in MyPlan 1.0

Providing tailored feedback

Definition: providing information related to the outcome of interest and based on a personal assessment [1]

Implementation in 'MyPlan 1.0'

Users complete the International Physical Activity Questionnaire (IPAQ) or the Flemish Fruit Test or the Flemish Vegetable Test in order to gain insight in their current behaviour. Then they receive an advice that compares their behaviour with the guidelines. Domains in which they can improve are explained (e.g. being active via active transport, eating fruit during breakfast, eating vegetables as snack) and examples on how they could improve are given.

Screenshot (translated to English):

Dear Louise,

You indicated that you eat fruit on **2 days per week** and that you eat **1 portion of fruit on average** on these days.

It is good that you eat fruit, since it is recommended to eat **two pieces of fruit a day** to achieve a healthy diet. Every step in the direction of this health standard can result in health benefits. **A good first step** for you could be to eat fruit on **more days** a week or to eat **more portions** of fruit.

To help you with this, you can create **your own plan** to eat more fruit via this website and the tips it offers. You will have complete control regarding on how many days you will eat fruit, how many portions of fruit you will eat and how you will do this. This way you will create **a personal and feasible fruit goal**. This advice can act as a guideline to determine your own fruit goal via your own plan.

To give you an idea of potential fruit goals you could set in your plan you can find some examples of goals that other people have chosen for their plans:

"Eric chose to eat one portion of fruit on 4 days a week"
"Ellen chose to eat two portions of fruit on 2 days a week"

Coping planning

Definition: identifying possible hindrances in the goal attainment process and deciding how to overcome these [2]

Implementation in 'MyPlan 1.0'

Users are provided with a list of possible hindrances, which they have to place in order of importance. They also have the chance to add their own barrier. After selecting the most applicable hindrance, they get to see predefined solutions for the hindrance, from which they can choose what they want to try themselves.

Screenshot (translated to English):

Please indicate which option describes best your most important barrier to eat more fruit.

If none of the options listed below fit, you can choose “other” and describe your barrier. If you do not experience any hindrances or barriers to eat more fruit, you can choose the last option.

Select one of the following options.

- I think many fruits are unappetizing
- I do not know many fruits
- I find fruit expensive
- I experience a lack of encouragement of my partner, family, friends, … to eat more fruits
- I find that fruits have a short shelf life
- I do not like to prepare fruit (washing, peeling, cutting, …)
- I do not know where I can eat fruits
- I find that there is not much fruit available at my workplace
- I find that there is not much fruit available at my home
- I often forget to eat fruit
- I often feel like eating something else than fruit
- None of the options listed above or other hindrances fit
- Other:

How would you like to eat more fruit?

You indicated that your most important barrier to eat more fruits is that **you do not like to prepare food (washing, peeling, cutting, …)**. Therefore, some methods are presented that might help you to eat more fruit. Select the method you would like to apply to eat more fruit. Choose a method that you perceive feasible to apply. You can also think up another method than the ones listed below and describe your method in the option “other”.

Select one of the following answers

- By also eating precut frozen fruit
- By also eating canned fruit (without added sugars)
- By preparing fruit in advance for several days (for example preparing a large bowl of fruit salad and keep it in the refrigerator)
- By selecting fruits that you do not have to prepare or that are easy to prepare (an unpeeled apple/pear, a kiwi cut in half, a banana, berries such as grapes, raspberries and cherries
- Other:

Action planning

Definition: specifying concretely when, where, and how you are going to achieve your goal [2]

Implementation in ‘MyPlan 1.0’

Users choose which behaviour they are going to perform and when and where exactly they are planning to do this. Users choose activities (e.g. certain sports), places (e.g. at work, at home) and moments (e.g. after work, during breakfast, …) from an extensive list of options, which they can extend with their own ideas. Thereafter, they have to state their plan in an implementation intention, formulated as an if-then sentence.

Screenshot (translated to English):

You chose to eat fruit on more days a week. You indicated that you now eat fruit on one day a week. Now you can set a goal by indicating on how many days you would like to eat fruit:

Select one of the following answers.

- ○ On 1 day a week
- ○ On 2 days a week
- ○ On 3 days a week
- ○ On 4 days a week
- ○ On 5 days a week
- ○ On 6 days a week
- ○ On 7 days a week

During which moments would you like to eat fruit?
(If you prefer another moment than the ones listed below, you can describe this in the option "other")

Multiple answers are possible.

- ❑ During breakfast
- ❑ As a snack in the morning
- ❑ During lunch time
- ❑ As a snack in the afternoon
- ❑ During dinner
- ❑ As a snack in the evening
- ❑ Other:

Self-monitoring of behaviour

Definition: keeping track of the specified goal behaviour [3]

Implementation in 'MyPlan 1.0'

The suggestion to monitor their behaviour change is made to users. Users can choose from different options how they are going to keep record of their behaviour (for example: in their agenda, via cellphone, …). Users can also tick the option that they prefer not to monitor their behaviour.

Screenshot (translated to English):

We advise you to monitor whether you follow your plan or not.

You can do this by writing down whether you have reached your goal and when you did so. Below you can find some proposals to do so.

Select the method you would like to apply to keep track of your goal on a daily basis. You can also think up your own method and describe it in the option "other".

- ○ I will monitor my goal via a booklet
- ○ I will monitor my goal via my calender
- ○ I will monitor my goal via my diary
- ○ I will monitor my goal using my computer (Excel, Word, online diary, …)
- ○ I will monitor my goal using my mobile phone (notes, calendar)
- ○ I will monitor my goal using an application on my smartphone or tablet
- ○ I would rather not monitor my goal
- ○ Other:

Social support

Definition: information that makes a person believe that he is liked, valued and part of a social network [4]

Implementation in 'MyPlan 1.0'

The feeling of social support is elicited by giving users the opportunity to e-mail their personal plan, not only to themselves, but also to a friend or family member.

<u>Screenshot (translated to English):</u>

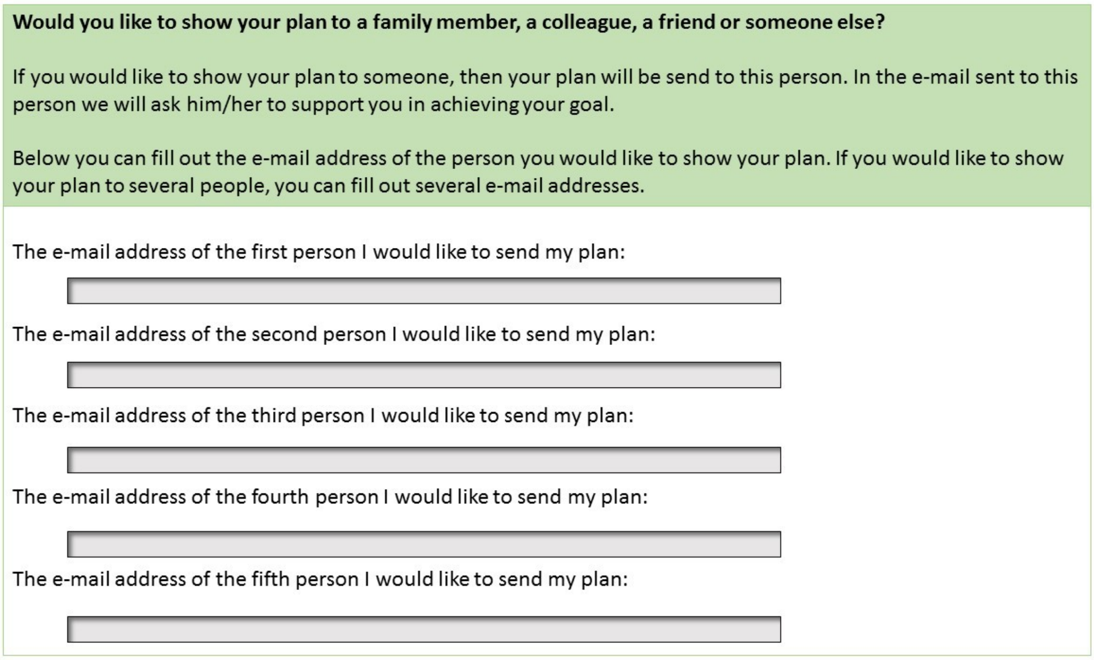

Would you like to show your plan to a family member, a colleague, a friend or someone else?

If you would like to show your plan to someone, then your plan will be send to this person. In the e-mail sent to this person we will ask him/her to support you in achieving your goal.

Below you can fill out the e-mail address of the person you would like to show your plan. If you would like to show your plan to several people, you can fill out several e-mail addresses.

The e-mail address of the first person I would like to send my plan:

The e-mail address of the second person I would like to send my plan:

The e-mail address of the third person I would like to send my plan:

The e-mail address of the fourth person I would like to send my plan:

The e-mail address of the fifth person I would like to send my plan:

References

1. Rimer BK, Kreuter MW. Advancing tailored health communication: A persuasion and message effects perspective. Journal of Communication; 2006; 56 (s1): S184–S201. DOI: 10.1111/j.1460-2466.2006.00289.x
2. Carraro N, Gaudreau P. Spontaneous and experimentally induced action planning and coping planning for physical activity: A meta-analysis. Psychology of Sport and Exercise; 2013; 14 (2): 228-248. DOI: 10.1016/j.psychsport.2012.10.004
3. Michie S, Ashford S, Sniehotta FF, Dombrowski SU, Bishop A, French DP. A refined taxonomy of behaviour change techniques to help people change their physical activity and healthy eating behaviours: The CALORE-taxonomy. Psychology and Health; 2011; 26 (11); 1479–1498. DOI: 10.1080/08870446.2010.540664
4. Cobb SMD. Social Support as a Moderator of Life Stress. Psychosomatic Medicine: 1976; 38(5), 300-314
